# GLP-1 receptor agonist therapy and skeletal muscle: A narrative review synthesizing adult evidence with implications for older adults

**DOI:** 10.1016/j.jarlif.2026.100083

**Published:** 2026-08-14

**Authors:** Krish Jagasia, Andrew M. Pfeiffer, Kenneth Vitale

**Affiliations:** a Division of Geriatrics, Gerontology and Palliative Care, University of California San Diego School of Medicine, 9500 Gilman Drive, La Jolla, CA, 92093, USA; b University of California San Diego School of Medicine, 9500 Gilman Drive, La Jolla, CA, 92093, USA; c Department of Orthopedic Surgery, University of California San Diego School of Medicine, 9500 Gilman Drive, La Jolla, CA, 92093, USA

**Keywords:** GLP-1 receptor agonist, Older adults, Skeletal muscle, Sarcopenia, Frailty, Obesity

## Abstract

Glucagon-Like peptide-1 receptor agonists (GLP-1-RAs) are increasingly prescribed for type 2 diabetes mellitus and chronic weight management, especially for the rapidly-growing population of older adults. However, older adults are also at heightened risk for sarcopenia and frailty, raising morbidity concerns about GLP-1-RA-induced lean body mass loss. This narrative review summarizes recent studies estimating skeletal muscle mass and/or volume, strength, composition, performance, and acute physiological responses during GLP-1-RA therapy in humans, with particular attention to implications for older adults. Because direct evidence in older adults is scarce, this review primarily synthesizes data from general adult populations and reasonably extrapolates implications for older adults. Most studies found that GLP-1-RA therapy was associated with a loss of skeletal muscle mass, accounting for up to 8.5% to 24.5% of total weight loss. Studies assessing muscle quality mostly saw improved muscle quality following GLP-1-RA therapy due to higher muscle density and decreased fat content. Studies measuring muscle strength generally found no significant change. However, from 21 included studies, only four studies specifically examined the older adult population, being methodologically heterogeneous and difficult to compare. The limited published literature in older adults provides insufficient evidence to determine age-related vulnerability to skeletal muscle loss while on GLP-1-RA and the potential clinical complications of resultant frailty; additional research in older adult populations is needed. Provisional considerations for preserving muscle mass during GLP-1-RA therapy are discussed, with particular relevance to older adults. This review demonstrates that the degree of skeletal muscle loss is variable but can account for a meaningful fraction of weight lost during therapy. Providers should consider these potential effects when prescribing GLP-1-RAs, especially in older adults at risk for frailty.

## Highlights

• GLP-1-RA therapy is associated with a loss of skeletal muscle mass in most studies, generally without significant changes in strength.
• GLP-1-RA therapy may lead to increased muscle quality.
• Studies on GLP-1-RA therapy in older adults are limited and more research is needed.
• Providers prescribing GLP-1-RAs should be aware of potential muscle loss, particularly in older adults at risk for sarcopenia.
• Monitoring muscle health, exercise, and diet are critical during GLP-1-RA therapy.

## 1 Introduction

### 1.1 Background

Glucagon-like peptide-1 receptor agonists (GLP-1-RAs) are a class of medications that exert their effects by activating GLP-1 receptors and mimic endogenous GLP-1. Their effects include stimulating insulin release, inhibiting glucagon release, delaying gastric emptying, and suppressing appetite. These effects led to their initial approval for the treatment of type 2 diabetes mellitus (T2DM) in 2005 [1,2]. Given their ability to promote weight loss, GLP-1-RAs have increasingly been approved and used for chronic weight management since 2015 [3].

Beyond glycemic control and weight loss, there are expanding indications suggesting that GLP-1-RAs have potential pleiotropic effects, including treatment of cardiovascular disease (CVD), heart failure with preserved ejection fraction (HFpEF), chronic kidney disease (CKD), metabolic dysfunction-associated steatohepatitis (MASH), obstructive sleep apnea (OSA), and peripheral artery disease (PAD), among others [1,3]. Emerging data further suggest that GLP-1-RA usage is associated with a lower risk of all-cause dementia and current research is examining whether it slows cognitive decline [1]. As of October 2025, twelve GLP-1-RA therapies have been approved for treatment of T2DM or obesity by the United States Food and Drug Administration (FDA), with >40 agents currently in development [3].

### 1.2 The older adult and GLP-1-RA

A population of interest for the use of GLP-1-RA therapy is older adults, typically defined as aged ≥65. This group represents the fastest-growing demographic in the world, but also the most sedentary, spending an estimated 9.7 h per day engaged in sedentary behavior [[4], [5], [6]]. Sedentary behavior is robustly associated with T2DM and weight gain, which may partly explain why half of the individuals with T2DM are older adults and 38.9% of older adults are obese [[7], [8], [9], [10], [11]]. Furthermore, the prevalence of T2DM is expected to dramatically increase over the next few decades due to the aging population and increasing numbers of higher-risk groups [12]. Concurrently, GLP-1-RA use rose from 3.4% to 13.8% in older adults with T2DM from 2017 to 2022 [13].

Despite the expanding indications for GLP-1-RAs, some studies report concerns that GLP-1-RA therapy is associated with the loss of skeletal muscle [14,15]. This is hypothesized to occur through appetite suppression, leading to reduced caloric (including protein) intake and triggering of catabolic hormones utilizing muscle mass for energy [16]. These concerns are most relevant for older adults at the highest risk of sarcopenia, estimated to affect 18.8% of community-dwelling older adults [17]. In older adults with diabetes, sarcopenia prevalence increases to 28.5%, with a threefold higher risk of sarcopenia compared with non-diabetics [18,19]. Sarcopenia can lead to major clinical morbidity and mortality due to weakness, poor balance, and difficulty with daily tasks, a 1.6-fold increase in fall risk and a 1.7–1.8-fold increase in fracture risk [20,21]. These effects can be devastating for older adults, especially in cases of hip fracture. For instance, hip fractures in older adults often lead to significant and permanent declines in physical and psychological quality of life, with most patients never returning to pre-fracture levels of independence [22].

There are additional considerations that make older adults a group of significant interest for GLP-1-RA treatment. Notably, older adults are more likely to have concurrent comorbidities that can complicate treatment. Approximately 30% of older adults with diabetes have coronary heart disease, twice the rate observed in non-diabetics [12]. Older adults also have a high prevalence of hypertension (HTN), CVD, heart failure, and CKD, many of which are emerging indications of GLP-1-RA treatment [12,23]. In fact, early research shows GLP-1-RAs can provide both cardiovascular and renal benefits, with studies estimating a 14% reduction in major adverse cardiovascular events (MACE) and a 14% reduction in kidney events [24,25]. This suggests multiple use cases for GLP-1-RA in this population. However, due to the likelihood of having multiple comorbidities, polypharmacy can be widespread in older adults, with one study estimating that 46% of older adults with diabetes have polypharmacy (defined as the use of ≥ 5 medications) [26]. Importantly, polypharmacy is associated with increased adverse drug events, hypoglycemia, drug-drug interactions, and reduced adherence [26,27]. One study estimates that 55% of older adults with diabetes are prescribed at least one potentially inappropriate medication [27]. As GLP-1-RA’s potential downstream effects and polypharmacy’s negative effects are not commonly discussed in the literature, such considerations should be important to physicians balancing the benefits and risks of GLP-1-RA treatment in older adults.

A recent meta-analysis of randomized controlled trials found that GLP-1-RA therapy led to, on average, roughly 28% of total weight loss being attributable to muscle-based indices [28]. While this suggests an emerging pattern of GLP-1-RA-induced muscle loss, direct evidence in older adults remains minimal, and the functional consequences of this muscle loss (i.e. effects on strength, gait, falls, frailty, and independence) are largely unknown in this population. Critically, the endpoints most commonly reported in GLP-1-RA trials (i.e. total weight loss, change in body mass index (BMI), single time-point lean mass estimation) are insufficient to capture dimensions of performance which are most relevant for older adults. For instance, BMI may misclassify body composition in older adults due to age-related shifts in body fat and muscle distribution, while total weight loss cannot distinguish between the loss of fat and lean mass [29]. Additionally, single time-point estimations of lean mass do not capture the trajectories of muscle quality or performance (e.g. strength, gait, frailty), which are the outcomes that most directly determine functional independence and survival in older populations [30,31]. This gap between the available evidence and clinical needs of older adults serves as the rationale for the present review and its performance-based framing.

## 2 Methods and aims

Despite the growing body of evidence on GLP-1-RAs and body composition, significant gaps remain in the literature. Most existing reviews are difficult to interpret due to the confounding effects of different measurement methods and amalgamation of human and animal studies [32]. For instance, many studies use lean mass as a proxy for muscle mass, making them difficult to compare with studies that estimate muscle mass with other methods. Studies also differ in the dose titration of the GLP-1-RA medication used, making it essential to analyze this factor as a source of variability. Critically, older adults are severely underrepresented in clinical trials of GLP-1-RAs. This underrepresentation currently limits the ability to make informed treatment decisions for a population at high risk of sarcopenia and functional decline. Therefore, the goal of this narrative review is to summarize the most recent literature on GLP-1-RAs and muscle health in adult populations, with particular attention to implications for older adults. By doing so, this review aims to clarify the currently scattered evidence on the effects of GLP-1-RAs on muscle, thereby providing provisional considerations for maintaining muscle health during GLP-1-RA therapy, with particular relevance to older adults.

As a narrative review, this work does not provide pooled estimates or draw causal inference, but aims to identify patterns, gaps, and clinically relevant signals within heterogeneous literature. A literature search was conducted in January 2026 using PubMed and Google Scholar. The search strategy combined three conceptual domains: 1) GLP-1-RA-related terms (e.g. “GLP-1″, “GLP-1 receptor agonist”, “semaglutide”, “liraglutide”, “tirzepatide”), 2) muscle-related terms (e.g. “muscle mass”, “muscle volume”, “muscle loss”, “skeletal muscle”), and 3) contextual terms (e.g. “body composition”, “older adults”, and “sarcopenia”). In order to maximize article retrieval, various combinations of terms from each domain were used. For example, representative PubMed search strings included (“GLP-1″ OR “GLP-1 receptor agonist” OR “semaglutide” OR “liraglutide” OR “tirzepatide”) AND (“muscle mass” OR “muscle volume” OR “muscle loss” OR “skeletal muscle”) AND (“body composition” OR “older adults” OR “sarcopenia”). The search was not restricted by language. Reference lists of retrieved articles were also hand-searched to identify additional studies. The authors screened article titles and abstracts for relevance, followed by full-text review to determine study eligibility.

Articles were included if they: 1) were published within the last seven years (2020–2026), 2) reported at least one outcome in one or more of the following four domains: (a) muscle quantity via estimation of skeletal muscle mass or volume (i.e. BIA, MRT, CT, or administrative diagnosis codes), (b) muscle composition or quality (i.e. intramuscular fat content, muscle density or attenuation, or muscle quality indices), (c) muscle strength or physical performance (i.e. handgrip strength, gait speed, chair rise time, six-minute walk distance), or (d) acute mechanistic responses to GLP-1 or GLP-1-RA exposure (i.e. muscle protein synthesis, microvascular blood flow), and 3) were conducted in human participants. Studies were excluded if they 1) were published before 2020, 2) were conducted in animal models, 3) only reported lean body mass, and 4) did not report original data. By excluding animal studies and studies using proxies for skeletal muscle mass (i.e. lean mass), this review aims to minimize confounders and synthesize studies with direct relevance to researchers and clinicians. We included human studies reporting estimated or proxy measures of skeletal muscle quantity (e.g. BIA, MRI/CT volumes, clinical diagnosis codes), muscle composition or density, muscle strength or performance (e.g. handgrip strength, six-minute walk), and acute physiological responses to GLP-1 or GLP-1-RA exposure in order to capture the full breadth of available evidence on the effects of GLP-1 and GLP-1-RAs on skeletal muscle. Furthermore, to our knowledge, this is the first review to separate studies of general adults from older adults to allow for potential comparison.

## 3 Literature review

### 3.1 GLP-1-RAs and muscle in general populations

#### 3.1.1 Decreased muscle mass (Bioelectrical impedance analysis)

Most studies used bioelectrical impedance analysis (BIA), a popular yet indirect method of measuring body composition utilizing the resistance from electrical current to estimate muscle mass [33]. While used frequently, it creates an estimate of lean mass. Beginning with studies using semaglutide, a retrospective observational cohort study by Chun et al. [14], showed a 4.11 kg mean weight loss and 0.88 kg decrease in skeletal muscle mass, suggesting that 21.4% of the weight lost was from skeletal muscle. However, the study also noted that the proportion of skeletal muscle mass relative to total mass actually increased [34]. Similarly, Xiang et al., 2023 (also a retrospective observational cohort study) showed a mean weight loss of 9.9 kg and 4.8% decrease in skeletal muscle mass [35]. Changes in handgrip strength were not significant. In a prospective single-arm study, Volpe et al., 2022 examined the effects of semaglutide in participants with T2DM, finding that skeletal muscle mass decreased by 1.3 kg/m^2^, but changes in handgrip strength or muscle quality were not significant [36]. A retrospective observational cohort study by Ozeki et al., 2022 evaluated participants with obesity and T2DM [37]. On average, participants lost 3.1 kg, their BMI decreased by 1.2 kg/m^2^, and their skeletal muscle mass decreased by 0.5 kg, suggesting that skeletal muscle mass accounted for 16.1% of the weight lost. While the mass percentage of skeletal muscle increased by 0.4%, this was only marginally significant (p = 0.06).

Several other studies used liraglutide. An uncontrolled pre-post study by Anesto & Nicolau, 2024 assessed the impact of liraglutide on participants without diabetes. Mean weight loss was 14.99 kg, of which 2.02 kg was muscle, accounting for 13.5% of weight loss [38]. In the retrospective observational cohort study by Keskin & Yaprak, 2022, participants with T2DM and obesity underwent treatment with either liraglutide and metformin, or just metformin [39]. In the liraglutide and metformin group, participants lost 11.4 kg of total body weight, lost 0.97 kg of muscle mass (8.5% of total weight lost), and had a 6.41 kg/m^2^ decrease in BMI. Meanwhile, in the metformin-only group, participants lost 4.6 kg of total body weight, lost 2.05 kg of muscle mass (44.6% of total weight lost), and had a 2.1 kg/m^2^ decrease in BMI. Interestingly, this study suggested that in comparison to metformin alone, liraglutide increased weight loss while preserving muscle mass.

One prospective single-arm study by Yamaguchi et al., 2025 used tirzepatide on participants with T2DM, showing that participants lost 4.9 kg of total body weight, of which 1.2 kg was from skeletal muscle mass (24.5% of total weight loss) [40]. Taken together, these studies demonstrate a consistent trend of skeletal muscle mass loss from GLP-1-RA therapy, with their variability in outcomes potentially due to differences in patient demographics (i.e. sex, age) and/or study design (i.e. type of GLP-1-RA, length of treatment). Notably, the majority of these studies were retrospective observational cohorts or prospective single-arm studies without control groups, suggesting that the reported within-group changes should be interpreted as observed associations rather than causal treatment effects.

#### 3.1.2 Decreased muscle volume (Other methods)

Studies that did not use BIA either used magnetic resonance imaging (MRI), computed tomography (CT), or ICD codes to determine muscle loss. A prospective single-arm study by Ditzenberger et al., 2025 used MRI to analyze the psoas muscle in participants with HIV treated with semaglutide and observed a 9.3% decrease in psoas muscle volume and mean weight loss of 7.8 kg [41]. However, psoas muscle fat, chair rise time, and gait speed did not change significantly. A retrospective observational cohort study by Nelson et al., 2024 examined muscle mass with CT-based AI deep learning [42]. Participants underwent semaglutide treatment and had a mean weight loss of 1.1 kg, with a 3.5 cm^2^ decrease in psoas muscle area. Additionally, muscle attenuation decreased by 3.4 Hounsfield Units (HU), suggesting a decrease in muscle density and/or quality. Finally, Butsch et al., 2025 (a retrospective observational cohort study) used ICD codes to determine muscle loss in participants who underwent GLP-1-RA treatment and found that 3% of participants were diagnosed with muscle loss (based on use of “muscle loss M62.5, M62.81–84″ codes) within one year of starting GLP-1-RA therapy [43]. While these three studies show a decrease in muscle volume, differences in measurement techniques make it difficult to draw conclusions regarding outcomes, and the use of observational or single-arm designs lacking control groups necessitate cautious interpretation of within-group changes which may not represent causal treatment effects. The studies observing muscle loss following GLP-1-RA treatment are shown in Table 1 and summarized by subject demographics, study methods, and outcomes.

**Table 1:** GLP-1 Therapy and Muscle Loss.

Authors, Year	Study Type	Participants	Controls	Baseline Frailty Status	Average Age (Age Range)	% Male	GLP-1-RA, Duration, and Dosage	Type of Muscle	Muscle Outcome Domains	Muscle Effects	BMI/Weight
Chun et al., 2025	Retrospective Observational Cohort	308 participants from clinical weight management program	N/A	Not reported	46.6 (23–73)	14%	semaglutide, three months, 0.25 mg administered weekly and titrated to a maximum dosage of 2.4 mg	skeletal muscle assessed by InBody 570	Quantity	skeletal muscle mass decreased by 0.88 kg but, proportion of skeletal muscle mass to total mass increased	mean weight loss 4.11 kg
Xiang et al., 2023	Retrospective Observational Cohort	43 participants with obesity	N/A	Not reported	30.4 (Not reported)	23%	semaglutide, six months, 0.25 mg administered weekly and titrated to a maximum dosage of 1.0 mg	skeletal muscle assessed by InBody S10	Quantity Strength/ Performance	skeletal muscle mass decreased by 4.8% changes in handgrip strength were not significant	mean weight loss was 9.9 kg
Volpe et al., 2022	Prospective Single-Arm	48 participants with T2DM	N/A	Not reported	57.7 (42–73)	54.2%	semaglutide, 52 weeks, 0.25 mg administered weekly and titrated to a maximum dosage of 1.0 mg	skeletal muscle assessed by SMF-BIA	Quantity Composition/ Quality Strength/ Performance	skeletal muscle mass decreased by 1.3 kg/m^2^ changes in handgrip strength and muscle quality were not significant	Not reported
Ozeki et al., 2022	Retrospective Observational Cohort	13 participants with obesity and T2DM	N/A	Not reported	52.0 (Not reported)	Not reported	semaglutide, three months, dosage not reported	skeletal muscle assessed by InBody 770	Quantity	skeletal muscle mass decreased by 0.5 kg but, skeletal muscle mass percentage did not change significantly (p = 0.06), and actually increased	mean weight loss was 3.1 kg mean BMI decrease was 1.2 kg/m^2^
Anesto & Nicolau, 2024	Uncontrolled Pre-Post Study	67 participants without diabetes	N/A	Not reported	46.8 (Not reported)	20.9%	liraglutide, eight months, 3 mg	skeletal muscle assessed by Tanita BC-420MA	Quantity	muscle mass decreased by 2.02 kg	mean weight loss was 14.99 kg (13.04%)
Keskin & Yaprak, 2022	Retrospective Observational Cohort	276 participants with T2DM and obesity	N/A	Not reported	49.7 (29–69)	31.9%	liraglutide and/or metformin, 12 weeks, 3 mg liraglutide administered daily and titrated to a maximum dosage of 6 mg	skeletal muscle assessed by Tanita BC-420MA	Quantity	liraglutide and metformin group lost 0.97 kg of muscle mass metformin-only group lost 2.05 kg of muscle mass	mean weight loss was 11.4 kg in liraglutide and metformin group mean BMI decrease was 6.41 kg/m^2^ in liraglutide and metformin group mean weight loss was 4.6 kg in metformin-only group mean BMI decrease was 2.1 kg/m^2^ in metformin-only group
Yamaguchi et al., 2025	Prospective Single-Arm	16 participants with T2DM	N/A	Not reported	54.3 (20–74)	81.2%	tirzepatide, 12 weeks, 2.5 mg administered weekly and titrated to a maximum dosage of 5.0 mg	skeletal muscle assessed by InBody S19	Quantity	skeletal muscle mass decreased by 1.2 kg (1.8%)	mean weight loss 4.9 kg
Ditzenberger et al., 2025	Prospective Single-Arm	46 participants with HIV and MASLD	N/A	Not reported	50 (Not reported)	63%	semaglutide, 24 weeks, 0.25 mg administered weekly and titrated to a maximum dosage of 1.0 mg	psoas muscle assessed by MRI	Quantity Composition/ Quality Strength/ Performance	psoas muscle volume decreased by 9.3% psoas muscle fat, time to rise from chair, and gait speed did not change	mean weight loss 7.8 kg
Nelson et al., 2024	Retrospective Observational Cohort	241 participants with CT scan data	N/A	Not reported	60.4 (Not reported)	37.3%	semaglutide, dosage not reported	skeletal muscle mass assessed by CT-based AI deep learning	Quantity Composition/ Quality	muscle area decreased by 3.5 cm^2^ muscle attenuation decreased by 3.4 HU	mean weight loss was 1.1 kg
Butsch et al., 2025	Retrospective Observational Cohort	461,382 adults from Inovalon Insights Claims Data	N/A	Not reported	52.9 (18–89)	43.7%	variable GLP-1-RA, one year, variable dosage	muscle loss identified based on diagnosis codes	Quantity	3% of participants diagnosed with muscle loss within a year of starting GLP-1-RA therapy	Not reported

#### 3.1.3 No effect

Despite many of the above studies reporting loss of skeletal muscle mass or volume after GLP-1-RA treatment, some studies did not find any effect. A different prospective single-arm study by Volpe et al., 2022 assessed the impact of semaglutide on participants with T2DM using BIA to evaluate skeletal muscle mass, observing that changes in skeletal muscle mass, muscle quality, and handgrip strength were not significant [44]. The retrospective observational cohort study by Wang et al., 2025 used either semaglutide, liraglutide, lixisenatide, beinaglutide, exenatide, dulaglutide, or PEG-loxenatide for 12 months. Skeletal muscle mass was also assessed by BIA, but changes in skeletal muscle mass were not significant [45]. Lastly, Uchiyama et al., 2023 (a retrospective observational cohort study) examined participants with T2DM on semaglutide for 24 weeks, assessed skeletal muscle mass with BIA [46]. Participants had an average BMI decrease of 1.3 kg/m^2^, but changes in skeletal muscle mass were not significant. These three studies illustrate that GLP-1-RA treatment does not always result in the loss of skeletal muscle mass, suggesting that other unmeasured variables (i.e. clinical comorbidities, baseline physical activity levels, nutritional intake) may influence outcomes.

#### 3.1.4 Muscular benefits

Amidst studies reporting the loss or preservation of muscle mass following GLP-1-RA treatment, some research suggests that GLP-1-RAs lead to increased muscle quality due to decreased fat content or improvements in strength and/or endurance measures. A randomized, open-label, parallel-group, phase 3 trial by Sattar et al., 2025 evaluated the impact of tirzepatide or insulin degludec on muscle volume and fat infiltration based on thigh muscle MRI measurements [47]. The tirzepatide group showed decreased muscle fat infiltration but also decreased muscle volume, with an average of 9.6 kg total body weight loss and mean BMI decrease of 3.4 kg/m^2^. In comparison, the insulin degludec group had increased muscle volume with no effect on muscle fat infiltration, a mean weight gain of 3.2 kg, and mean BMI increase of 1.1 kg/m^2^. Similar results are reported by the randomized, double-blind, placebo-controlled trial by Pandey et al., 2024, which used liraglutide vs. placebo to assess thigh muscle with MRI [48]. They found that the liraglutide group lost 2.87% of muscle fat, but also lost 3.18% of muscle volume. Additionally, the percentage of participants with “adverse muscle composition” (defined as muscle with high fat content and low volume) decreased from 11.0% to 8.2% in the liraglutide group. None of these changes were observed in the placebo group. In a prospective, single-arm study, Kakegawa et al., 2024 examined the impact of semaglutide on participants with both T2DM and metabolic dysfunction-associated steatotic liver disease (MASLD) [49]. Participants underwent MRI to measure psoas, paraspinal, and abdominal muscles; skeletal muscle steatosis fraction decreased from 12.8 to 9.9, indicating less fat content. Changes in skeletal muscle mass or mean weight loss were not significant. Lastly, a randomized, double-blind, placebo-controlled trial by Kosiborod et al., 2023 measured 6-minute walk distance for participants receiving semaglutide vs. placebo, all of whom had HFpEF [50]. The semaglutide group had a mean weight loss of 13.3% and demonstrated a 21.5 m increase in 6-minute walk distance, while the placebo group had a mean weight loss of 2.6% and demonstrated a 1.2 m increase in 6-minute walk distance. Generally, these studies demonstrate that muscle quality and muscle volume are separate measurements that can be differentially impacted by GLP-1-RA treatment, highlighting the need to assess muscle holistically. Among the studies reporting muscular benefits, Sattar et al., 2025, Pandey et al., 2024, and Kosiborod et al., 2023, include randomized comparator groups, providing stronger evidence for between-group comparisons and causal treatment effects. In comparison, the within-group changes in Kakegawa et al., 2024 (a prospective single-arm study) should be interpreted as observed associations, and are not comparable to between-group changes. The studies observing preserved or improved muscle following GLP-1-RA treatment are shown in Table 2.

**Table 2:** GLP-1 Therapy: Preserved or Improved Muscle Outcomes.

Authors, Year	Study Type	Participants	Controls	Baseline Frailty Status	Average Age (Age Range)	% Male	GLP-1-RA, Duration, and Dosage	Type of Muscle	Muscle Outcome Domains	Muscle Effects	BMI/Weight
Volpe et al., 2022	Prospective Single-Arm	40 participants with T2DM	N/A	Not reported	64.9 (Not reported)	52.5%	semaglutide, 26 weeks, 0.25 mg administered weekly and titrated to a maximum dosage of 1.0 mg	skeletal muscle assessed by SMF-BIA	Quantity Composition/ Quality Strength/ Performance	changes in skeletal muscle mass, muscle quality, and handgrip strength were not significant	Not reported
Wang et al., 2025	Retrospective Observational Cohort	679 participants from an obesity and weight loss clinic	N/A	Not reported	37 (Not reported)	30.8%	variable GLP-1-RA, 12 months, variable dosage	skeletal muscle assessed by InBody	Quantity	changes in skeletal muscle mass were not significant	Not reported
Uchiyama et al., 2023	Retrospective Observational Cohort	25 participants with T2DM	N/A	Not reported	54.1 (28–78)	56%	semaglutide, 24 weeks, 3 mg oral taken daily and titrated to a maximum dosage of 14 mg	skeletal muscle assessed by BIA (Tanita MC-780MA-N)	Quantity	changes in skeletal muscle mass were not significant	mean BMI decrease was 1.3 kg/m^2^
Sattar et al., 2025	Randomized, Open-Label, Parallel-Group, Phase 3 Trial	172 participants with T2DM receiving tirzepatide	55 participants with T2DM receiving insulin degludec	Not reported	56.0 (Not reported)	59.8%	tirzepatide or insulin degludec, 52 weeks, with tirzepatide 2.5 mg administered weekly and titrated to a maximum dosage of 5.0 mg, 10 mg, or 15 mg	thigh muscle assessed by MRI	Quantity Composition/ Quality	tirzepatide group had decreased muscle fat infiltration and decreased muscle volume insulin degludec group had increased muscle volume, but no significant effect on muscle fat infiltration	mean weight loss was 9.6 kg in tirzepatide group mean BMI decrease was 3.4 kg/m^2^ in tirzepatide group mean weight gain was 3.2 kg in insulin degludec group mean BMI increase was 1.1 kg/m^2^ in insulin degludec group
Pandey et al., 2024	Randomized, Double-Blind, Placebo-Controlled Trial	73 participants with overweight or obesity receiving liraglutide	55 participants with overweight or obesity receiving a placebo	Not reported	49.7 (Not reported)	7.8%	liraglutide, 40 weeks, 0.6 mg liraglutide administered daily and titrated to a maximum dosage of 3 mg	thigh muscle assessed by MRI	Quantity Composition/ Quality	liraglutide group had decreased muscle fat by 2.87% liraglutide group had reduced muscle volume of 3.18% liraglutide group decreased proportion of participants with adverse muscle composition (11.0% to 8.2%)	Not reported
Kakegawa et al., 2024	Prospective Single-Arm	21 participants with MASLD and T2DM	N/A	Not reported	52 (Not reported)	53.4%	semaglutide, six months, 0.25 mg administered weekly and titrated to a maximum dosage of 1.0 mg	psoas, paraspinal, abdominal wall muscles assessed by MRI	Quantity Composition/ Quality	skeletal muscle mass steatosis fraction decreased from 12.8 to 9.9 changes in skeletal muscle mass were not significant	mean weight loss was not significant
Kosiborod et al., 2023	Randomized, Double-Blind, Placebo-Controlled Trial	263 participants with HFpEF receiving semaglutide	266 participants with HFpEF receiving a placebo	Not reported	69 (Not reported)	43.9%	semaglutide, 52 weeks, 0.25 mg administered weekly and titrated to a maximum dosage of 2.4 mg	6-minute walk distance	Strength/ Performance	semaglutide group had 21.5 m increase in 6-minute walk distance, while placebo had 1.2 m increase	mean weight loss was 13.3% in semaglutide group mean weight loss was 2.6% in placebo group

### 3.2 GLP-1-RAs and muscle in older adults

The literature on the effect of GLP-1-RAs on muscle in older adults (defined as adults aged ≥65) is much more scarce, and only four studies met inclusion criteria for this review. One retrospective observational cohort study by Ren et al., 2025 evaluated the effectiveness of semaglutide for 24 months in participants aged ≥65 with T2DM (average age of 72.6 years and 49.1% male), with one group receiving semaglutide and another group as a control [51]. Skeletal muscle was assessed with BIA. In the group receiving semaglutide, the mean decrease in BMI was 2.79 kg/m^2^ in males and 3.25 kg/m^2^ in females. Muscle mass decreased by 0.41 kg/m^2^ in males (14.7% of total weight lost) and 0.31 kg/m^2^ in females (9.5% of total weight lost). Handgrip strength decreased (1.45 kg in males and 1.46 kg in females) in addition to gait speed (0.05 m/s in males and 0.04 m/s in females). Another retrospective observational cohort study by Osaka et al., 2023 studied participants aged ≥70 (average age of 76.8 years and 60% male) taking either a GLP-1-RA and basal insulin, or just basal insulin [52]. Skeletal muscle was assessed with BIA. Interestingly, skeletal muscle mass increased by 0.78 kg in the group receiving GLP-1-RA and basal insulin, while skeletal muscle mass changes were not significant in the group receiving only basal insulin. Two similar acute experimental studies by Abdulla et al., 2020 and Abdulla et al., 2023 evaluated the short-term effects of GLP-1 infusions (3 h) on eight men aged ≥65 with an average age of 71 [53,54]. In these studies, leg muscle microvascular and macrovascular blood flow were measured with ultrasound. In Abdulla et al., 2020, GLP-1 was found to increase muscle protein synthesis by 0.044 percent/hour. In Abdulla et al., 2023, GLP-1 increased muscle microvascular blood flow five-fold and also increased whole-body glucose uptake by 5.4 mg/kg^−1^/180 min^−1^. The studies observing impacts of GLP-1-RA treatment in older adults are shown in Table 3.

**Table 3:** GLP-1 Therapy in Older Adults: Limited and Heterogeneous Evidence.

Authors, Year	Study Type	Participants	Controls	Baseline Frailty Status	Average Age (Age Range)	% Male	GLP-1-RA, Duration, and Dosage	Type of Muscle	Muscle Outcome Domains	Muscle Effects	BMI/Weight
Ren et al., 2025	Retrospective Observational Cohort	220 participants aged ≥65 with T2DM receiving semaglutide	212 controls aged ≥65 with T2DM	27.7% of participants had sarcopenia	72.6 (Not reported)	49.1%	semaglutide, 24 months, variable dosage	skeletal muscle assessed by bioelectric impedance analysis BCA-1C	Quantity Strength/ Performance	semaglutide group had decreased muscle mass (0.31 kg/m^2^ in females and 0.41 kg/m^2^ in males) semaglutide group had decreased handgrip strength (1.46 kg in females and 1.45 kg in males) and gait speed (0.04 m/s in females and 0.05 m/s in males)	mean BMI decrease was 3.25 kg/m^2^ in females mean BMI decrease was 2.79 kg/m^2^ in males
Osaka et al., 2023	Retrospective Observational Cohort	10 participants aged ≥70 receiving GLP-1-RA and basal insulin	10 participants aged ≥70 receiving basal insulin	Not reported	76.8 (Not reported)	60%	either a variable GLP-1-RA and basal insulin, or just basal insulin, variable dosage	skeletal muscle assessed by InBody 770	Quantity	skeletal muscle mass increased by 0.78 kg in group receiving GLP-1-RA and basal insulin skeletal muscle mass change in the basal insulin group was not significant	Not reported
Abdulla et al., 2020	Acute Experiment	8 men aged ≥65	Same participants, different ocassion	Not reported	71 (65–75)	100%	GLP-1 infusion, 3 h, 1.2 pmol/kg^−1^/min^−1^	leg muscle micro and macrovascular blood flow were assessed with ultrasound	Acute Mechanistic Responses	leg muscle protein synthesis increased by 0.044 percent/hour	Not reported
Abdulla et al., 2023	Acute Experiment	8 men aged ≥65	Same participants, different ocassion	Not reported	71 (65–75)	100%	GLP-1 infusion, 3 h, 1.2 pmol/kg^−1^/min^−1^	leg muscle micro and macrovascular blood flow were assessed with ultrasound	Acute Mechanistic Responses	leg muscle microvascular blood flow increased five-fold whole-body glucose update increased by 5.4 mg/kg^−1^/180 min^−1^	Not reported

### 3.3 Summary of findings

In order to facilitate interpretation across the heterogeneous evidence base, the 21 included studies can be categorized by the muscle outcome domains (quantity, quality/composition, strength/performance, and acute mechanistic responses) that were assessed. Regarding muscle quantity, most studies estimated muscle mass with BIA [[34], [35], [36], [37], [38], [39], [40],[44], [45], [46],^51^,^52^], while some studies measured muscle volume with MRI or CT [41,42,[47], [48], [49]]. One study (Butsch et al., 2025) used administrative diagnosis codes as a proxy for muscle loss [43]. Studies assessing muscle quality/composition included Ditzenberger et al., 2025, Kakegawa et al., 2024, Nelson et al., 2024, Pandey et al., 2024, Sattar et al., 2025, and Volpe et al., 2022 (both studies) [36,41,42,44,[47], [48], [49]]. Studies assessing muscle strength/performance included Ren et al., 2025, Volpe et al., 2022 (both studies), and Xiang et al., 2023 [35,36,44,51]. One study (Kosiborod et al., 2025) only assessed physical performance with walking speed [50]. Lastly, the two studies by Abdulla et al., 2020 and Abdulla et al., 2023 assessed acute mechanistic responses (i.e. muscle protein synthesis, microvascular blood flow) to native GLP-1 infusion [53,54], and therefore provide short-term physiological evidence of native GLP-1 as opposed to long-term clinical evidence of GLP-1-RAs. From this categorization, it is clear that most of the included studies focused on muscle mass or volume, leaving evidence on muscle strength, performance, composition, or mechanistic responses to be more sparse.

A consistent but heterogeneous pattern emerges across the 21 studies evaluating GLP-1-RA therapy and skeletal muscle outcomes. Table 1, Table 2, Table 3 summarize the characteristics of the 21 studies on GLP-1-RAs and skeletal muscle effects, stratifying them thematically to highlight where evidence is strong, mixed, and missing. Taken together, the literature on GLP-1-RAs and impacts on skeletal muscle in the general population suggests that muscle loss can account for a meaningful portion of total weight loss from GLP-1-RA therapy, ranging from 8.5% to 24.5% for the studies included in this review. In general populations, 12 studies reported a loss of skeletal muscle mass and/or volume, while four studies reported no change.

Results on measures of muscle strength and quality were less conclusive, but generally suggested that changes in muscle strength were not significant, while muscle quality (based on either intramuscular fat content and/or various measures of muscle strength or performance) improved. However, these studies remain difficult to standardize. While two studies found no changes in handgrip strength and one study found no changes in chair rise time or gait speed [35,41,44], these represent very different exercise parameters. And, while the study by Kosiborod et al., 2023 saw an increase in 6-minute walk distance, this outcome may be due to weight loss and does not necessarily indicate increased muscular strength [50]. The majority of studies observed decreased intramuscular fat content, suggesting potentially improved muscle quality and relatively greater density [[47], [48], [49]]. Still, conflicting data exists. One study found that changes in muscle quality (defined by calculating hand grip strength divided by skeletal muscle mass) were not significant[44], and another reported that changes in muscle fat content were not significant [41]. Interestingly, one study even found a decrease in muscle density and/or increased fat content in participants [42].

Of the 21 studies, only four studies specifically focused on the older adult population aged ≥65. Furthermore, because two of these studies were acute experiments only measuring short-term effects of GLP-1 (and via infusion), they are difficult to compare with other studies assessing long-term effects of injectable GLP-1-RA treatment through designs such as observational cohorts, prospective single-arm studies, and randomized trials [53,54]. Hence, only two retrospective observational cohort studies (Ren et al., 2025 and Osaka et al., 2023) remained available for direct comparison of older adults vs. general populations, and even these studies were heterogeneous in design [51,52]. The study by Ren et al. observed that skeletal muscle mass decreased after GLP-1-RA treatment in older adults, while the study by Osaka et al. found that skeletal muscle mass increased only when a GLP-1-RA was taken with basal insulin, making direct conclusions difficult. Therefore, there is no conclusive evidence suggesting that GLP-1-RA treatment leads to unique effects in older adults. Because the literature on the effects of GLP-1-RAs in older adults is very scarce and potential side effects of sarcopenia can be detrimental, more studies are needed for determining age-specific risk-benefit assessments for older adults considering GLP-1-RA treatment.

An important distinction should be drawn between absolute muscle loss observed during GLP-1-RA-induced weight loss and clinically meaningful sarcopenia or functional decline. Weight loss from any intervention (i.e. caloric restriction, bariatric surgery) is often accompanied by some degree of lean mass loss, which is a normal physiological response and does not always reflect a pathological process [55]. The definition of sarcopenia by the European Working Group on Sarcopenia in Older People (EWGSOP2) describes a muscular disease primarily characterized by low muscular strength, with low muscle quantity/quality providing evidence to support the diagnosis and poor physical performance indicating severity [30]. By this definition, the muscle mass reductions reported in most studies in this review do not necessarily constitute sarcopenia, as they were often not accompanied by clinically significant declines in strength or physical performance in the studies assessing those outcomes. Only the study in older adults by Ren et al., 2025, reported concurrent declines in muscle mass, handgrip strength, and gait speed, which is more consistent with a trajectory toward sarcopenia but insufficient on its own to conclude that GLP-1-RA treatment in older adults increases sarcopenia risk [51]. Whether the muscle loss often observed during GLP-1-RA therapy crosses the threshold into clinically meaningful sarcopenia or functional decline remains an unanswered question requiring longer-term studies with validated functional endpoints.

Beyond potential changes in skeletal muscle mass and quality, GLP-1-RA therapy should be interpreted through a performance-based framework asking if and how therapy changes the trajectory of functional aging. Among older adults, changes in muscle mass and quality are most significant when they affect functional outcomes such as strength, gait speed, balance, activities of daily living (ADLs), falls, recovery after injury, and independence [56]. Current definitions of sarcopenia support this concept by focusing on low muscle strength as opposed to mass, classifying sarcopenia as severe when reductions in muscle mass or quality are accompanied by impaired physical performance [30]. This framing aligns with the frailty phenotype involving three or more of the following criteria: unintentional weight loss, exhaustion, weakness, slow gait speed, and low physical activity [57]. Within this framework, muscle changes associated with GLP-1-RAs should be studied as potential modifiers of physical functioning over time, not simply changes in mass. It should be emphasized that the potential effects of GLP-1-RA-associated muscle loss on falls, frailty, resilience, and independence remain hypothesized and have not been directly measured in the included studies. These functional consequences are extrapolated from the broader geriatric literature on sarcopenia and should be interpreted as theoretical, unlike the strictly measured outcomes (muscle mass, volume, strength, and composition) reported in the reviewed studies. While it is possible that GLP-1-RA therapy could worsen physical functioning in susceptible older adults who lose significant skeletal muscle mass, are inactive, and/or lack adequate nutritional intake, therapy may also improve performance by reducing adiposity and mechanical load from excess body fat. This uncertainty necessitates that future longitudinal studies explore the impact of GLP-1-RA on trajectories of physical functioning, resilience, and recovery. Existing longitudinal studies show that functional outcomes such as grip strength and gait speed are clinically meaningful predictors of healthy aging and survival, suggesting the value of their incorporation into future longitudinal studies of GLP-1-RAs to assess performance trajectories [30,31]. Modifiable lifestyle factors should also be considered, given that current studies in obese older adults show that the combination of aerobic and resistance training during non-GLP-1-RA-induced weight loss improves functional outcomes more effectively than either modality alone [58]. Clinically, these findings support a shift from focusing on muscle mass alone to evaluating how GLP-1-RA therapy alters functional trajectories, particularly in older adults at risk of sarcopenia and frailty.

### 3.4 Conceptual framework

The evidence synthesized in this review points toward a central construct that can be termed *GLP-1-RA-associated body composition shift as a trade-off relevant to performance*. This construct postulates that GLP-1-RA therapy redistributes body composition in ways that may reduce overall adiposity while simultaneously decreasing muscle quantity, with differential and sometimes opposing effects on muscle composition/quality and physical strength/performance. We hypothesize that this trade-off may be especially significant for older adults where even modest reductions in muscle mass may accelerate trajectories toward sarcopenia, frailty, and loss of independence, while reductions in adiposity can improve mobility and cardiometabolic health. From an operational perspective, this framework distinguishes three dimensions that may shift together or apart during GLP-1-RA therapy: 1) muscle quantity (mass or volume), 2) muscle quality (composition, density, intramuscular fat content), and 3) physical performance (strength, gait speed, endurance, balance, and ADLs). The reviewed evidence suggests that during GLP-1-RA therapy in general adult populations, muscle quantity often decreases, muscle quantity may improve (through reduced intramuscular fat), and muscle strength is often preserved in the short term, although these dimensions have rarely been assessed simultaneously and almost never in older adults over clinically meaningful time periods. This framework would be falsified or substantially limited if carefully controlled longitudinal studies in older adults demonstrated that lean mass reductions during GLP-1-RA therapy are consistently accompanied by preserved or improved physical performance, without an increase in sarcopenia risk, falls, frailty, or loss of independence. Conversely, if longitudinal data showed that GLP-1-RA-associated muscle loss in older adults is accompanied by declining functional trajectories, even when muscle quality improves, this framework would be strengthened.

It remains important to consider that the type and strength of evidence supporting each dimension of this framework varies considerably. Randomized trials with comparator groups (Kosiborod et al., 2023; Pandey et al., 2024; Sattar et al., 2025) can support cautious statements about treatment effects on muscle quality and functional endpoints [47,48,50]. In contrast, single-arm prospective studies and observational studies, which constitute the majority of the evidence base on GLP-1-RAs and muscle, should be interpreted as demonstrating associations rather than causal treatment effects. The extrapolation from measured outcomes (muscle quantity, quality/composition, and strength/performance) to hypothesized effects on performance (frailty, falls, and independence) remains speculative and requires direct testing in future longitudinal studies of older adults.

### 3.5 Potential morbidity and mortality of sarcopenia in older adults

The functional implications of body composition changes due to GLP-1-RA use in older adults are incompletely understood, but potentially severe. Shorter clinical trials (≤6 months) of GLP-1-RAs in general adult populations frequently report preserved handgrip strength even when lean mass is lost, while longer observational studies (≥12 months) in older adults with T2DM are more likely to suggest a decline in strength [59]. This discrepancy may reflect the fact that shorter trials (3–6 months) can less reliably appreciate the loss of skeletal muscle in comparison to longer trials lasting 12–24 months. It is also possible that improvements in muscle quality reported by some studies may offset the initial reductions in muscle mass, although reductions in functional capabilities may become more apparent as treatment progresses.

Older adults are at heightened risk for overall functional decline resulting from decreases in strength while on GLP-1-RA therapy. This is supported by the study from Ren et al., 2025, showing the long-term (24 month) effects of semaglutide in older adults with reductions in handgrip strength and gait speed [51]. Importantly, reductions in hand grip strength are well-known to be strongly associated with increased all-cause mortality, cardiovascular mortality, and morbidity across multiple large prospective cohort studies [[60], [61], [62]]. Beyond mortality, reduced grip strength further predicts functional decline, hospitalization, and overall disability. In one study, each 5 kg decrease in grip strength was associated with increased odds of ADL limitations ranging from 6% for toileting to 20% for eating [63]. In the oldest old (≥80 years), low grip strength independently predicts mortality, hospitalization, and disability onset [64]. These results were obtained even after adjusting for muscle mass, inflammatory markers, and comorbidities. The critical implications of decreased grip strength while on GLP-1-RA highlight the necessity for future studies to examine older adult populations to validate the findings from Ren et al., 2025 [51].

Additionally, a recent review found no clear evidence that GLP-1-RA therapy enhanced cardiorespiratory fitness [65]. These observations make it seem less likely that the increase in 6-minute walk distance from the Kosiborod et al., 2023 study was purely due to increased strength or cardiovascular fitness, and could more likely be a result of reductions in mechanical load from weight loss [50]. Because older adults are more likely to experience difficulty performing ADLs independently, the distinction between muscle mass and function while on GLP-1-RA further mandates the need for more studies examining functional outcomes (i.e. strength testing, gait speed, cardiorespiratory fitness) and standardization of measurement, rather than relying solely on body composition analysis.

### 3.6 Critical analysis of study heterogeneity and methodology

The substantial variability in reported muscle loss parameters across studies may be attributed to several key methodological and population differences. First, the method of assessing muscle mass or volume has inherent limitations. Although BIA is widely available and provides practical advantages including low cost, portability, and ease of repeated measurements, it provides an estimate (not a direct measurement) of muscle mass and results can be less reliable in individuals with obesity due to excess intramuscular lipids and adipose tissue [66]. It is also significantly influenced by hydration status, time of day, and recent urination [67,68]. It should be noted that dual-energy X-ray absorptiometry (DXA) remains the reference standard for body composition assessment and sarcopenia diagnosis, offering superior accuracy for estimating appendicular lean mass relative to BIA [30]. However, DXA is often cost-prohibitive and inaccessible for routine longitudinal tracking in outpatient weight-loss settings, while BIA provides a more pragmatic, portable, and low-cost alternative for body composition assessments, leading many studies to utilize BIA for these reasons [69]. Crucially, studies comparing BIA and DXA show strong correlations (r = 0.94–0.97) for estimating appendicular skeletal muscle mass [69]. It is important to note that BIA can systematically overestimate lean mass, potentially leading to underdiagnosis of low muscle mass if DXA-derived cutoffs are applied [70]. The predominance of BIA-based studies in this review reflects the practical realities of clinical research in weight management settings, although it remains important to note that BIA-derived estimates may not be directly comparable to DXA-derived values.

By contrast, MRI or CT directly measure muscle volume and can assess metrics of muscle quality (i.e. muscle density, intramuscular fat) which BIA cannot capture, leading to more reliable results at the expense of cost burden and accessibility in some settings [66,71]. This may partially explain why the studies in this review using BIA reported more variable results on skeletal muscle mass loss and the degree of loss. Specifically, three studies using BIA found no muscular effect of GLP-1-RA therapy [[44], [45], [46]], one study found skeletal muscle accounted for 8.5% of total weight loss [39], and another found skeletal muscle accounted for 24.5% of total weight loss [40]. Studies using MRI or CT more consistently reported a loss in skeletal muscle volume but are less common potentially due to cost and accessibility [41,42]. The variation in measurement complicates the ability to make direct comparisons between studies, and standardization of skeletal muscle mass measurement would greatly improve understanding of GLP-1-RA’s effects on skeletal muscle.

Another important source of heterogeneity is variation in GLP-1-RA dosing across the included studies. As recommended by the American Gastroenterological Association (AGA), standard dose titration schedules call for subcutaneous semaglutide to be initiated at 0.25 mg weekly and titrated every four weeks to a maximum dosage of 2.4 mg weekly for obesity or 1.0–2.0 mg weekly for T2DM; subcutaneous liraglutide is recommended to be initiated at 0.6 mg daily and titrated weekly to a maximum dosage of 3.0 mg daily for obesity or 1.8 mg daily for T2DM; and subcutaneous tirzepatide to be initiated at 2.5 mg weekly and titrated every four weeks to a maximum dosage of 15 mg weekly [72,73]. Importantly, clinical judgment is essential for adjusting the titration schedule based on individual patient tolerance, given that some patients may achieve strong responses at submaximal doses, allowing for the long-term continuation of that dose [73].

Among the included studies, while all subcutaneous semaglutide studies used standard titration schedules starting at 0.25 mg weekly, five studies[35,36,41,44,49]. titrated to a maximum dose of 1.0 mg (recommended for T2DM), two studies [34,50]. titrated to a maximum dose of 2.4 mg (recommended for obesity), and the rest did not report dosage or had variable doses [37,42,51]. For subcutaneous liraglutide, one study [48]. followed standard guidelines of 0.6 mg daily titrated to 3.0 mg, one study [38]. used a fixed dose of 3.0 mg daily (the maximum obesity dose), while another study [39]. started from 3.0 mg daily and exceeded the recommended maximum dose to reach 6.0 mg daily. For the two studies using subcutaneous tirzepatide, both started with the standard 2.5 mg weekly dose but one [40]. only titrated to 5.0 mg (well below the standard maximum dose of 15.0 mg), and another [47]. had variable maximum doses of 5.0 mg, 10.0 mg, or 15.0 mg. The remaining studies used variable GLP-1-RAs or native GLP-1 infusion, which are not comparable to standard GLP-1-RA pharmacotherapy [43,45,[52], [53], [54]]. These dosing differences may partly explain the heterogeneity in muscle outcomes. A recent network meta-analysis found that GLP-1-RA regimens with higher doses (e.g. semaglutide 2.4 mg, tirzepatide 15 mg) achieved greater total weight loss but were the least effective in preserving lean mass [74]. In the present review, the limited number of studies and wide heterogeneity makes it difficult to observe whether such patterns exist, although they remain important for consideration. Notably, no age-specific dosing guidelines currently exist for GLP-1-RAs in older adults, although the American Diabetes Association (ADA) Standards of Care recommend slower titration and monitoring for excessive weight loss and sarcopenia in this population [75]. Whether lower maintenance doses could preserve muscle mass while still achieving therapeutic benefit in older adults remains an important question for future investigation.

Beyond dosing differences, variation in individual tolerance to GLP-1-RA therapy represents an additional and underappreciated source of variability. The most common adverse effects of GLP-1-RAs are gastrointestinal side effects (i.e. nausea, vomiting, diarrhea, constipation), often occurring during initiation and dose escalation [76]. Indeed, approximately 6–10% of patients in clinical trials of GLP-1-RAs permanently discontinue therapy due to adverse effects [76], illustrating the importance of considering individual tolerance to GLP-1-RAs. Critically, however, none of the included studies in this review systematically reported data on gastrointestinal tolerance, dose reductions due to side effects, or the relationship between tolerance and muscle outcomes. The absence of this data represents a significant limitation; while speculative, these side effects may reduce caloric or protein intake, potentially affecting muscle loss or leading to reductions in dosage or treatment discontinuation. Future studies should aim to directly test this hypothesis.

Importantly, baseline prevalence of sarcopenia or frailty was only reported by one study [51]. The effects of GLP-1-RA treatment may change across populations with different baseline levels of muscle mass, making this an important consideration for comparisons across studies. Because it is hypothesized that GLP-1-RA therapy may increase the risk of sarcopenia, future studies should aim to collect such data in order to assess whether this premise holds true [15].

The presence or absence of T2DM among participants may also influence outcomes. A recent meta-analysis found that GLP-1-RAs induce significant reductions in muscle mass among non-diabetic populations (−1.41 kg; p < 0.001), but not among individuals with T2DM (−0.74 kg; p = 0.10) [77]. The reason for this effect is unknown, but potentially due to a higher baseline muscle mass in non-diabetics, muscle-protective effects (improved glycemic control and insulin sensitivity) in diabetic populations, or both [77,78]. While there are no consistent differences in outcomes between studies of participants with and without T2DM in this review, this factor remains an important consideration when interpreting GLP-1-RA trials.

Sex differences also warrant consideration. Older postmenopausal women with declining estrogen levels experience fat redistribution which increases the risk of obesity and T2DM [79], complicating GLP-1-RA effects on obesity and body mass measurements. A recent meta-analysis found that GLP-1-RA treatment showed greater weight loss in women than men, with sex differences becoming more apparent as more weight was lost [80]. While the outcomes of the studies in this review did not clearly differentiate based on participant sex, it remains an important factor for better understanding the heterogeneity of the results. Importantly, none of the included studies that enrolled postmenopausal women reported or controlled for the use of sex hormone therapy (HT). A large cross-sectional study of postmenopausal women found that prolonged HT use was associated with higher appendicular lean mass and a lower prevalence of sarcopenia [81]. Therefore, HT following menopause may provide a modest protective effect on muscle mass. However, a systematic review of 43 studies found no consistent evidence that HT benefits sarcopenia-related outcomes, noting a high risk of bias among the included studies [82]. Based on a meta-analysis of 12 randomized trials, the overall effect of HT on lean body mass appears to be small and not clinically significant for the average postmenopausal woman [83]. However, because HT status was not reported in any of the included studies, it remains unknown whether concurrent HT use may have influenced the observed muscle outcomes in postmenopausal participants in the context of GLP-1-RAs. Given its effect on muscle mass, future studies examining GLP-1-RA effects on muscle in women should account for menopausal status and HT use, as the lack of such data may currently limit interpretation.

Lastly, baseline physical activity status and nutritional intake (both important determinants of muscle preservation) were rarely reported in the reviewed studies. Lean mass retention has been shown to be heavily influenced by the amount of caloric restriction, rate of weight loss, and presence or absence of structured resistance training [84]. Yet, obtaining accurate measures of nutritional intake and physical activity can be extremely challenging in study design. In the few cases where these factors were reported in the included studies, the inconsistency in measuring these variables represents a considerable limitation for interpreting whether observed muscle loss may be an unavoidable consequence of GLP-1-RA therapy, or whether it is largely shaped by other lifestyle factors.

## 4 Clinical implications for practice, with particular relevance to older adults

The observed association between GLP-1-RA therapy and decreased skeletal muscle mass in several studies has important implications for clinical practice, particularly for older adults. Older adults face unique vulnerabilities, such as substantial risk for functional decline, falls and fall-related complications, and loss of independence, all of which demand attention to GLP-1-RA therapies in these populations [85]. Despite these risks, existing literature suggests that adverse outcomes of GLP-1-RA treatment among older adults may be mitigated with four key strategies: monitoring muscle health, nutritional interventions, exercise interventions, and adjunct pharmacotherapies. Critically, these strategies should not be interpreted as prescriptive clinical protocols, but rather, provisional considerations that should be individualized for each patient’s unique clinical context (i.e. renal function, nutritional status, comorbidities, contraindications).

First, measuring and monitoring muscle health during GLP-1-RA therapy can help identify potential declines and allow providers to intervene early. Grip strength is traditionally the preferred standard to measure muscle health in older adults, but because the studies in this review rarely observed significant changes in grip strength during GLP-1-RA therapy, other methods could also be considered [86]. Regular evaluations of 6-minute walking distance and gait speed may be beneficial in older adults at risk of frailty and falls, which the loss of skeletal muscle mass could exacerbate [[87], [88], [89]]. While no studies have yet shown that GLP-1-RA treatment directly increases sarcopenia risk in older adults specifically, it remains a significant concern [90]. While DXA remains the reference standard for body composition assessment in the sarcopenia literature, routine DXA monitoring during GLP-1-RA therapy has not been validated and may not be practical in most clinical settings.

A second strategy for preserving muscle health during treatment is adequate nutrition. Consensus guidelines for older adults recommend consuming at least 1.0–1.2 g of protein per kilogram of body weight daily for healthy older adults [91,92]. However, in the specific context of GLP-1-RA therapy for obesity, a recent joint advisory recommends 1.2–1.6 g of protein per kilogram body weight daily to preserve muscle mass [55]. Protein source is another important consideration. Complete proteins should be prioritized, and a recent study found that the dietary requirement of leucine in older adults was roughly 78 mg/kg/day, which is higher than most current recommendations [93]. Critically, this leucine threshold is taken from the general nutrition and aging literature and, to our knowledge, has not been studied specifically in the context of GLP-1-RA therapy. Plant-based protein, dairy, lean poultry, seafood, and eggs tend to be encouraged in comparison to red and processed meats [55]. Older adults should also ensure adequate levels of vitamin D, which is essential for maintaining high bone mineral density in a population at risk of osteoporosis and fractures, although this recommendation is taken from the general nutrition and aging literature as opposed to being specific for GLP-1-RA therapy [15]. Vitamin D deficiencies are common worldwide, especially among older adults, and may independently represent another risk factor for sarcopenia [94]. A recent consensus statement recommends combining vitamin D supplementation with calcium in order to reduce fracture risk in older adults [95]. Some studies also suggest that omega-3 fatty acids (such as eicosapentaenoic acid (EPA) and docosahexaenoic acid (DHA)) are beneficial at reducing sarcopenia risk, supporting the potential use of fish oil supplementation in older adults receiving GLP-1-RA therapy, although these recommendations also come from the general literature and future studies need to examine them in the context of GLP-1-RA therapy [15].

Third, exercise interventions are essential during treatment because protein intake alone is not sufficient to preserve muscle mass without resistance training [55]. An important consideration for starting an exercise regimen in patients receiving GLP-1-RA therapy is potential fatigue during treatment (a commonly reported side effect) often resulting from sedentary lifestyles, especially older adults. This may require slowly building up over time to a goal of 60 to 90 min of resistance training per week [14]. Incorporating aerobic exercise alongside resistance training is also beneficial, but no studies to our knowledge have examined the effects of exercise in the specific context of GLP-1-RA therapy. Weight regain after the completion of GLP-1-RA treatment is common, and continuing aerobic exercise and overall increased energy expenditure may help mitigate this risk [14]. Among older adults, it is essential that physical activity recommendations are patient-specific, as this population can have widely different fitness levels and comorbidities that influence their ability to exercise [15]. Some older adults may not be able to attain standard exercise recommendations, which can cause decreased motivation and reduced adherence. Therefore, incorporating short sessions of exercise and intermittent forms of physical activity is a promising approach for older adults [96].

Finally, adjunct pharmacotherapies may be considered in cases of significant muscle loss unresponsive to lifestyle interventions, although no such drugs are currently approved by the FDA for the treatment of sarcopenia [97,98]. These pharmacological interventions must be used with caution in older adults due to the potential of adverse side effects, comorbidities and polypharmacy; few studies evaluate their clinical efficacy specifically on older adults [99]. Investigational agents targeting muscle preservation are discussed in Section IV (“Future Directions”).

**Fig. 1:**
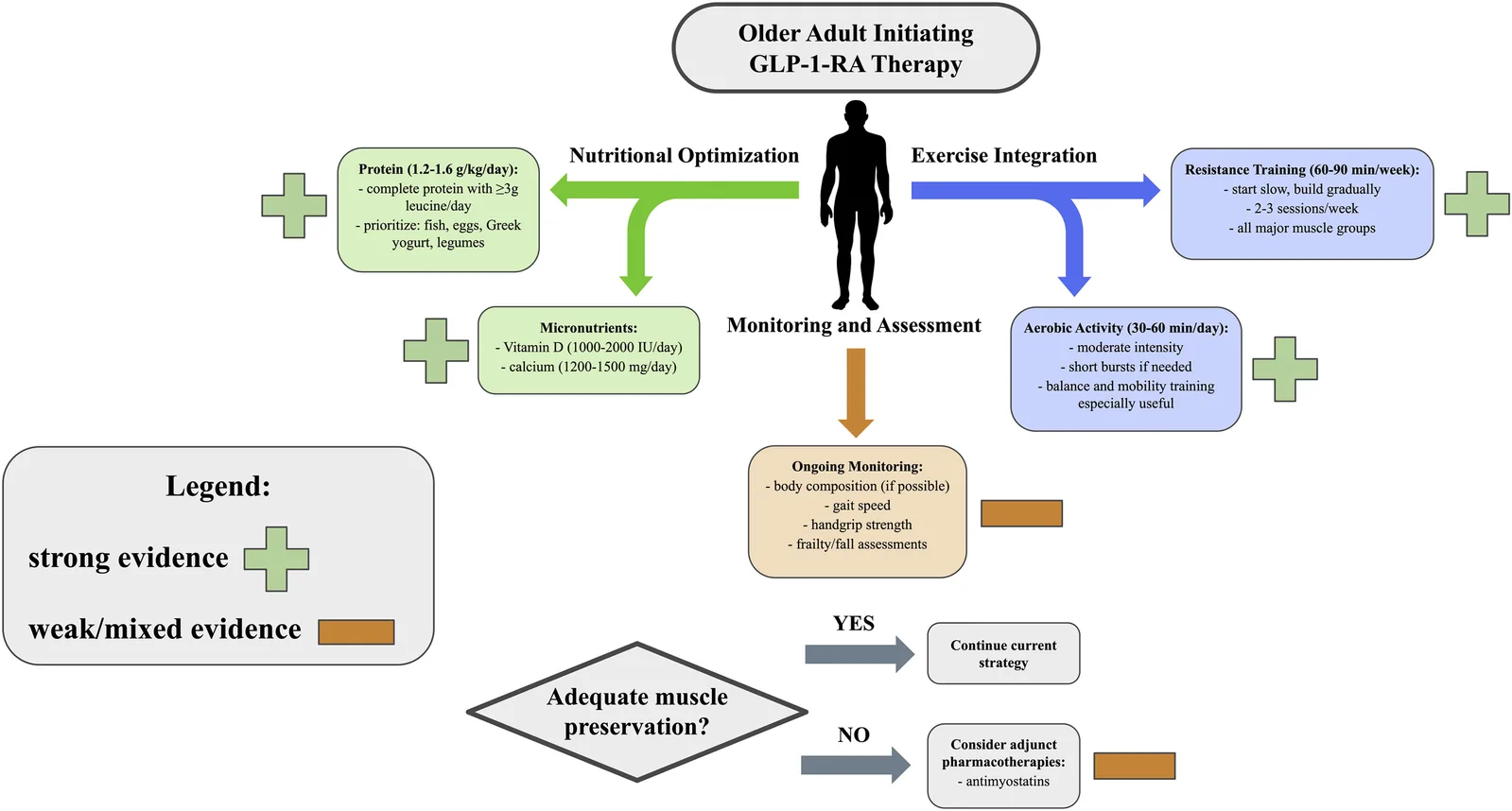
Provisional Multimodal Strategy for Preserving Muscle Health During GLP-1-RA Therapy in Older Adults.

Provisional considerations for preserving muscle health during GLP-1-RA therapy are summarized in Fig. 1, and a proposed monitoring framework is presented in Table 4. In this framework, three crucial clinical domains that warrant careful monitoring for older adults receiving GLP-1-RA treatment (muscle health and fall risk; nutritional status; functional capacity) are highlighted. Clinicians can reference the guidelines in this framework to perform baseline assessments, monitor status during GLP-1-RA therapy, and remain vigilant for potential red flags (see Table 4). These recommendations were not directly tested in the studies included in this review, and are instead derived from indirect evidence and general principles of sarcopenia prevention. Therefore, they should be regarded as hypothesis-generating rather than prescriptive clinical guidance. The individual medical context of each patient (i.e. renal function, nutritional status, comorbidities, contraindications) should always be considered when applying these suggestions. The specific thresholds and recommendations cited in Table 4 are drawn from the referenced literature in the table and are intended to serve as starting points for clinical decision-making as opposed to strict protocols.

**Table 4:** Proposed Monitoring Framework for Muscle Health During GLP-1 Therapy: Provisional Considerations Based on Indirect Evidence*.

Clinical Domain	Baseline Assessment	Monitoring During Therapy	Red Flags	References
**Muscle Health and Fall Risk** [59,100]	- fall history - SARC-F questionnaire	- fall diary - repeat SARC-F questionnaire each visit	- new difficulty with ADLs	- Marassi & Fadini, 2026 - Prokopidis, 2026
**Nutritional Status** [12,55]	- body weight - dietary protein intake - Vitamin D	- weight trajectory - protein intake logs - micronutrient levels every 6 months	- weight loss >2% per month - protein intake consistently <0.8 g/kg/day - Vitamin D deficiency	- LeRoith et al., 2019 - Mozaffarian et al., 2025
**Functional Capacity** [30,101,102]	- handgrip strength (HGS) - gait speed - ADL assessment	- repeat HGS every 3–6 months - repeat gait speed every 3–6 months	- large decline in HGS, HGS<16 kg in women, or HGS<27 kg in men⁎⁎ - gait speed decline >0.1 m/s in 12 months⁎⁎⁎ - new ADL dependence	- Adam et al., 2023 - Artaud et al., 2015 - Cruz-Jentoft et al., 2019

Footnotes:.

⁎ All thresholds and monitoring intervals in this table are provisional and performance-based, being adapted from the broader geriatric and sarcopenia literature cited in the References column and have not been validated specifically in the context of GLP-1-RA therapy. They are intended as provisional starting points for clinical decision-making, not as prescriptive protocols.

⁎⁎ Handgrip strength cutoffs are based on the EWGSOP2 consensus definition of sarcopenia.^30^ Alternative cutoffs have been proposed by the Sarcopenia Definitions and Outcomes Consortium (<20 kg in women, <35 kg in men) [103]. However, .all of these thresholds are derived from the general sarcopenia literature and have not been validated specifically in the context of GLP-1-RAs.

⁎⁎⁎ The threshold for gait speed is adapted from the geriatric literature on gait speed decline as a predictor of disability and falls and has not been derived from GLP-1-RA studies.

Fig. 1 Caption: Provisional considerations for preserving muscle health during GLP-1-RA therapy, with particular relevance to older adults. Monitoring and assessing muscle mass and/or function, nutritional optimization, and exercise integration may be considered, while adjunct pharmacotherapies remain investigational. A legend indicates where evidence for muscle-preserving strategies is strong and where it is weak or mixed. These considerations are provisional and performance-based, grounded primarily in indirect evidence from the literature on frailty, sarcopenia, nutrition, and general aging, as opposed to being derived from the GLP-1-RA trials themselves. They should not be interpreted as ready-to-implement clinical guidelines.

## 5 Future directions

While the available evidence suggests that GLP-1-RA therapy is frequently associated with some degree of skeletal muscle mass loss in general populations, studies specifically examining older adults are minimal, and the current evidence base is drawn predominantly from general adult populations. Specifically, age-specific guidelines on GLP-1-RA dosing, nutritional intake, and exercise habits during therapy would greatly aid clinicians prescribing GLP-1-RAs for older adults. Further, given that higher GLP-1-RA doses are associated with greater lean mass loss [74], future studies should investigate whether modified dose titration schedules or lower maintenance doses in older adults can balance therapeutic efficiency with muscle preservation. More studies examining functional outcomes (i.e. grip strength, gait speed) are especially important given the risk of falls and frailty in older adults. Furthermore, more longitudinal evidence is needed to determine the impact of GLP-1-RA therapy on performance-based trajectories of aging, such as physical function, resilience, and recovery. Future reviews on GLP-1-RAs and skeletal muscle should aim to stratify studies by methodological characteristics to minimize confounders. Alternatively, future studies can stratify by population characteristics, such as the presence or absence of T2DM and gender balance. Standardized muscle assessment methodologies are needed to allow for direct comparison between studies, given that results from BIA may not be comparable to results from MRI, CT, or DXA. The extent to which GLP-1-RA treatment may increase the risk of sarcopenia in older adults remains unknown.

Importantly, future studies can help determine whether nutritional and exercise interventions can preserve muscle mass in older adults receiving GLP-1-RAs, or if other strategies (i.e. adjunct pharmacotherapies) are needed. Several investigational pharmacological approaches may eventually complement lifestyle interventions for muscle preservation during GLP-1-RA therapy, although none are currently approved for this indication and none were examined in the studies included in this review. Notably, antimyostatins such as bimagrumab, landogrozumab, apitegromab, and taldefgrobep are an investigational class of drugs that function by blocking myostatin and other negative growth regulators of muscle. Early-phase trials suggest that these agents may preserve lean mass during GLP-1-RA-induced weight loss. For instance, results from the COURAGE trial suggest that the addition of the antimyostatin trevogrumab to semaglutide preserved 50–51% of lean mass compared with semaglutide alone [1]. However, these agents remain investigational, and larger and longer trials in the context of GLP-1-RA associated muscle loss are necessary to confirm efficacy and safety before they can be recommended for clinical use. Studies may also investigate combination therapy of GLP-1-RAs and muscle-preserving pharmacotherapies.

## 6 Conclusion

This narrative review primarily synthesizes evidence from general adult populations, as direct evidence on GLP-1-RA effects on skeletal muscle in older adults remains extremely limited, with only four of the 21 included studies specifically examining adults aged ≥65. The available evidence summarized in this narrative review suggests that GLP-1-RA therapy can be accompanied by skeletal muscle loss in some contexts, although findings may vary by population characteristics, drug and dosing, treatment duration, and measurement method, with several studies reporting preserved strength and muscle mass or improvements in muscle quality. Evidence for older adults specifically remains extremely limited, and the findings of this review are drawn predominantly from general adult populations with implications extrapolated to older adults. While the available data do not conclusively show which unique muscular effects of GLP-1-RAs exist in older adults, they indicate a pattern of potentially increased vulnerability to sarcopenia and frailty in this population. Due to a high baseline risk of sarcopenia, even modest muscle loss may carry greater functional consequences in older populations.

Given these findings, the widespread and expanding use of GLP-1-RAs suggests that clinicians should be aware of their effects on skeletal muscle mass and exercise caution in prescribing these medications for older adults. Clinicians should be proactive with patients to mitigate potential adverse outcomes by monitoring muscle health, recommending aerobic and resistance exercise training, and ensuring adequate protein and micronutrient intake. Ultimately, recognizing muscle health as a therapeutic priority is an important step toward safe and holistic patient care during GLP-1-RA treatment.

## Author contributions

All authors reviewed, edited, and approved the final manuscript.

## Data statement

Data sharing is not applicable to this narrative review article as no datasets were generated or analyzed during the current study.

## Funding sources

This research did not receive any specific grant from funding agencies in the public, commercial, or not-for-profit sectors.

## Declaration of generative AI and AI-assisted technologies in the writing process

No AI or AI-assisted technologies were used in the writing process or development of this manuscript.

## Ethical statement

Ethics approval and consent to participate were not required for this narrative review, as the study did not involve human participants or animal subjects.

## Declaration of competing interest

The authors declare that they have no known competing financial interests or personal relationships that could have appeared to influence the work reported in this paper.

## References

[1] Gonzalez-Rellan M.J., Drucker D.J. (2025). New molecules and indications for GLP-1 medicines. JAMA.

[2] Drucker D.J. (2024). Efficacy and safety of GLP-1 medicines for type 2 diabetes and obesity. Diabetes Care.

[3] Celletti F., Farrar J., De Regil L. (2025). World Health Organization guideline on the use and indications of glucagon-like peptide-1 therapies for the treatment of obesity in adults. JAMA.

[4] Gianfredi V., Nucci D., Pennisi F., Maggi S., Veronese N., Soysal P. (2025). Aging, longevity, and healthy aging: the public health approach. Aging Clin Exp Res.

[5] Raichlen D.A., Aslan D.H., Sayre M.K. (2023). Sedentary behavior and incident dementia among older adults. JAMA.

[6] Tadrous R., Forster A., Farrin A., Coventry P.A., Clegg A. (2025). Exploring the perceptions of sedentary behaviour in community-dwelling older adults aged 75 and older: a series of focus group interviews. BMC Public Health.

[7] Emmerich SD, D.V.M., Fryar CD, et al. Products - data briefs - number 508 September 2024. October 9, 2024. doi:10.15620/cdc/159281.

[8] Bellary S., Kyrou I., Brown J.E., Bailey C.J. (2021). Type 2 diabetes mellitus in older adults: clinical considerations and management. Nat Rev Endocrinol.

[9] Li X., Zhou T., Ma H., Liang Z., Fonseca V.A., Qi L. (2021). Replacement of sedentary behavior by various daily-life physical activities and structured exercises: genetic risk and incident type 2 diabetes. Diabetes Care.

[10] Silveira E.A., Mendonça C.R., Delpino F.M. (2022). Sedentary behavior, physical inactivity, abdominal obesity and obesity in adults and older adults: a systematic review and meta-analysis. Clin Nutr ESPEN.

[11] Wang L., Li X., Wang Z. (2021). Trends in prevalence of diabetes and control of risk factors in diabetes among US adults, 1999-2018. JAMA.

[12] LeRoith D., Biessels G.J., Braithwaite S.S. (2019). Treatment of diabetes in older adults: an Endocrine Society* clinical practice guideline. J Clin Endocrinol Metab.

[13] Zeyfang A., Gölz S., Iraci F. (2025). Pattern of antidiabetic drug prescription in older persons with type 2 diabetes: results from the German DPV registry. J Am Med Dir Assoc.

[14] Mehrtash F., Dushay J., Manson J.E. (2025). Integrating diet and physical activity when prescribing GLP-1s—Lifestyle factors remain crucial. JAMA Intern Med.

[15] Memel Z., Gold S.L., Pearlman M., Muratore A., Martindale R. (2025). Impact of GLP- 1 receptor agonist therapy in patients high risk for sarcopenia. Curr Nutr Rep.

[16] Mastaitis J.W., Gomez D., Raya J.G. (2025). GDF8 and activin A blockade protects against GLP-1–induced muscle loss while enhancing fat loss in obese male mice and non-human primates. Nat Commun.

[17] Wang L., Chao J., Zhang N. (2026). Prevalence and factors associated with sarcopenia in community-dwelling older adults: a systematic review and meta-analysis. Gerontology.

[18] Feng L., Gao Q., Hu K. (2022). Prevalence and risk factors of sarcopenia in patients with diabetes: a meta-analysis. J Clin Endocrinol Metab.

[19] Sazlina S.G., Lee P.Y., Chan Y.M., Hamid M.S.A., Tan N.C. (2020). The prevalence and factors associated with sarcopenia among community living elderly with type 2 diabetes mellitus in primary care clinics in Malaysia. Plos One.

[20] Yang C., Ye T., Gao Y. (2025). Association between sarcopenia and falls in Chinese older adults: findings from the China health and retirement longitudinal study. PloS One.

[21] Yeung S.S.Y., Reijnierse E.M., Pham V.K. (2019). Sarcopenia and its association with falls and fractures in older adults: a systematic review and meta-analysis. J Cachexia Sarcopenia Muscle.

[22] Alexiou K.I., Roushias A., Varitimidis S.E., Malizos K.N. (2018). Quality of life and psychological consequences in elderly patients after a hip fracture: a review. Clin Interv Aging.

[23] Fleg J.L., Forman D.E., Berra K. (2013). Secondary prevention of atherosclerotic cardiovascular disease in older adults. Circulation.

[24] Chen J.C., Fang Y.W., Liu Y.F., Chen M.T., Tsai M.H. (2026). GLP-1 receptor agonist therapy and cardiorenal outcomes in patients ≥ 80 years old with type 2 diabetes. J Am Geriatr Soc.

[25] Waqas S.A., Ali D., Afridi M.K. (2025). Efficacy of GLP-1 receptor agonists among older adults: a meta-analysis of cardio-kidney outcome trials. Arch Gerontol Geriatr.

[26] Remelli F., Ceresini M.G., Trevisan C., Noale M., Volpato S. (2022). Prevalence and impact of polypharmacy in older patients with type 2 diabetes. Aging Clin Exp Res.

[27] Lu L., Wang S., Chen J. (2023). Associated adverse health outcomes of polypharmacy and potentially inappropriate medications in community-dwelling older adults with diabetes. Front Pharmacol.

[28] Batsis J.A., Gavras A., Gross D.C. (2026). Effect of incretin-based and nonpharmacologic weight loss on body composition. Ann Intern Med.

[29] Ponti F., Santoro A., Mercatelli D. (2020). Aging and imaging assessment of body composition: from fat to facts. Front Endocrinol.

[30] Cruz-Jentoft A.J., Bahat G., Bauer J. (2019). Sarcopenia: revised European consensus on definition and diagnosis. Age Ageing.

[31] Studenski S., Perera S., Patel K. (2011). Gait speed and survival in older adults. JAMA.

[32] McMath A., Gallagher D. (2026). Understanding impact of anti-obesity medications on skeletal muscle mass change is confounded by measurement methods. *Obes Rev*. n/a(n/a).

[33] Ward L.C. (2019). Bioelectrical impedance analysis for body composition assessment: reflections on accuracy, clinical utility, and standardisation. Eur J Clin Nutr.

[34] Chun E., Siojo A., Rivera D. (2025). Weight loss and body composition after compounded semaglutide treatment in a real world setting. Diabetes Obes Metab.

[35] Xiang J., Ding X.Y., Zhang W. (2023). Clinical effectiveness of semaglutide on weight loss, body composition, and muscle strength in Chinese adults. Eur Rev Med Pharmacol Sci.

[36] Volpe S., Lisco G., Fanelli M. (2022). Once-weekly subcutaneous semaglutide improves fatty liver disease in patients with type 2 diabetes: a 52-week prospective real-life study. Nutrients.

[37] Ozeki Y., Masaki T., Kamata A. (2022). The effectiveness of GLP-1 receptor agonist semaglutide on body composition in elderly obese diabetic patients: a pilot study. Medicines.

[38] Blanco Anesto J., Nicolau J. (2024). [Changes in weight, body composition, metabolic parameters and vitamin D in subjects with grade 3 and 4 obesity treated with liraglutide 3 mg]. Nutr Hosp.

[39] Keskin L., Yaprak B. (2022). Comparison of the effect of liraglutide and metformin therapy on the disease regulation and weight loss in obese patients with type 2 diabetes mellitus. Eur Rev Med Pharmacol Sci.

[40] Yamaguchi Y., Kuwata H., Imura M. (2025). Early induction of insulin sensitisation treated by tirzepatide: a prospective, single-arm, open-label study in Japanese individuals with obesity and type 2 diabetes. Diabetologia.

[41] Ditzenberger G.L., Lake J.E., Kitch D.W. (2025). Effects of semaglutide on muscle structure and function in the SLIM LIVER study. Clin Infect Dis.

[42] Nelson L.W., Lee M.H., Garrett J.W. (2024). Intrapatient changes in CT-based body composition after initiation of semaglutide (Glucagon-Like Peptide-1 Receptor Agonist) therapy. Am J Roentgenol.

[43] Scott Butsch W., Sulo S., Chang A.T. (2025). Nutritional deficiencies and muscle loss in adults with type 2 diabetes using GLP-1 receptor agonists: a retrospective observational study. Obes Pillars.

[44] Volpe S., Lisco G., Racaniello D. (2022). Once-weekly semaglutide induces an early improvement in body composition in patients with type 2 diabetes: a 26-week prospective real-life study. Nutrients.

[45] Wang J., Lin C., Cai X. (2025). Glucagon-like peptide-1 receptor agonist treatment associated weight fluctuation and influencing factors in patients with overweight or obesity. Diabetes Obes Metab.

[46] Uchiyama S., Sada Y., Mihara S., Sasaki Y., Sone M., Tanaka Y. (2023). Oral semaglutide induces loss of body fat mass without affecting muscle mass in patients with type 2 diabetes. J Clin Med Res.

[47] Sattar N., Neeland I.J., Dahlqvist Leinhard O. (2025). Tirzepatide and muscle composition changes in people with type 2 diabetes (SURPASS-3 MRI): a post-hoc analysis of a randomised, open-label, parallel-group, phase 3 trial. Lancet Diabetes Endocrinol.

[48] Pandey A., Patel K.V., Segar M.W. (2024). Effect of liraglutide on thigh muscle fat and muscle composition in adults with overweight or obesity: results from a randomized clinical trial. J Cachexia Sarcopenia Muscle.

[49] Kakegawa T., Sugimoto K., Saito K. (2024). Favorable liver and skeletal muscle changes in patients with MASLD and T2DM receiving glucagon-like peptide-1 receptor agonist: a prospective cohort study. Medicine.

[50] Kosiborod M.N., Abildstrøm S.Z., Borlaug B.A. (2023). Semaglutide in patients with heart failure with preserved ejection fraction and obesity. N Engl J Med.

[51] Ren Q., Zhi L., Liu H. (2025). Semaglutide therapy and accelerated sarcopenia in older adults with type 2 diabetes: a 24-month retrospective cohort study. Drug Des Devel Ther.

[52] Osaka T., Hamaguchi M., Fukui M. (2023). Favorable appendicular skeletal muscle mass changes in older patients with type 2 diabetes receiving GLP-1 receptor agonist and Basal insulin Co-therapy. Clin Med Insights Endocrinol Diabetes.

[53] Abdulla H., Phillips B., Wilkinson D. (2023). Effects of GLP-1 infusion upon whole-body glucose uptake and skeletal muscle perfusion during fed-state in older men. J Clin Endocrinol Metab.

[54] Abdulla H., Phillips B.E., Wilkinson D.J. (2020). Glucagon-like peptide 1 infusions overcome anabolic resistance to feeding in older human muscle. Aging Cell.

[55] Mozaffarian D., Agarwal M., Aggarwal M. (2025). Nutritional priorities to support GLP-1 therapy for obesity: a joint Advisory from the American College of Lifestyle Medicine, the American Society for Nutrition, the Obesity Medicine Association, and The Obesity Society. Obesity.

[56] Lunt E., Ong T., Gordon A.L., Greenhaff P.L., Gladman JRF. (2021). The clinical usefulness of muscle mass and strength measures in older people: a systematic review. Age Ageing.

[57] Fried L.P., Tangen C.M., Walston J. (2001). Frailty in older adults: evidence for a phenotype. J Gerontol Ser A.

[58] Villareal D.T., Aguirre L., Gurney A.B. (2017). Aerobic or resistance exercise, or both, in dieting obese older adults. N Engl J Med.

[59] Prokopidis K. (2026). Glucagon-like peptide-1 receptor agonists and muscle strength changes in older adults: risks beyond muscle mass reductions. Br J Pharmacol.

[60] Celis-Morales C.A., Welsh P., Lyall D.M. (2018). Associations of grip strength with cardiovascular, respiratory, and cancer outcomes and all cause mortality: prospective cohort study of half a million UK Biobank participants. BMJ.

[61] López-Bueno R., Andersen L.L., Koyanagi A. (2022). Thresholds of handgrip strength for all-cause, cancer, and cardiovascular mortality: a systematic review with dose-response meta-analysis. Ageing Res Rev.

[62] Leong D.P., Teo K.K., Rangarajan S. (2015). Prognostic value of grip strength: findings from the Prospective Urban Rural Epidemiology (PURE) study. The Lancet.

[63] Mcgrath R.P., Vincent B.M., Lee I.M., Kraemer W.J., Peterson M.D. (2018). Handgrip strength, function, and mortality in older adults: a time-varying approach. Med Sci Sports Exerc.

[64] Legrand D., Vaes B., Matheï C., Adriaensen W., Van Pottelbergh G., Degryse J.M. (2014). Muscle strength and physical performance as predictors of mortality, hospitalization, and disability in the oldest old. J Am Geriatr Soc.

[65] Liu Z., Weeldreyer N.R., Angadi S.S. (2025). Incretin receptor agonism, fat-free mass, and cardiorespiratory fitness: a narrative review. J Clin Endocrinol Metab.

[66] Kiefer L.S., Fabian J., Rospleszcz S. (2022). Population-based cohort imaging: skeletal muscle mass by magnetic resonance imaging in correlation to bioelectrical-impedance analysis. J Cachexia Sarcopenia Muscle.

[67] Compher C., Cederholm T., Correia MITD (2022). Guidance for assessment of the muscle mass phenotypic criterion for the Global Leadership Initiative on Malnutrition diagnosis of malnutrition. J Parenter Enter Nutr.

[68] NIH Consensus statement (1996). Bioelectrical impedance analysis in body composition measurement. National Institutes of Health Technology Assessment Conference Statement. December 12-14, 1994. Nutrition.

[69] Liu J., Chen X. (2023). Comparison between bioelectrical impedance analyses and dual-energy X-ray absorptiometry for accuracy in assessing appendicular skeletal muscle mass and diagnosing sarcopenia in hospitalized Chinese older adults. Medicine.

[70] Achamrah N., Colange G., Delay J. (2018). Comparison of body composition assessment by DXA and BIA according to the body mass index: a retrospective study on 3655 measures. PloS One.

[71] Bosy-Westphal A., Jensen B., Braun W., Pourhassan M., Gallagher D., Müller M.J. (2017). Quantification of whole-body and segmental skeletal muscle mass using phase-sensitive 8-electrode medical bioelectrical impedance devices. Eur J Clin Nutr.

[72] Moiz A., Peters T.M., Tsoukas M.A., Yu O.H.Y., Filion K.B., Eisenberg M.J. (2026). Balancing the benefits and risks of GLP-1 receptor agonists: a clinical guide for shared decision-making. eClinicalMedicine.

[73] Grunvald E., Shah R., Hernaez R. (2022). AGA clinical practice guideline on pharmacological interventions for adults with obesity. Gastroenterology.

[74] Karakasis P., Patoulias D., Fragakis N., Mantzoros C.S. (2025). Effect of glucagon-like peptide-1 receptor agonists and co-agonists on body composition: systematic review and network meta-analysis. Metabolism.

[75] Older Adults (2026). Standards of care in diabetes—2026. Diabetes Care.

[76] Kushner R.F., Almandoz J.P., Rubino D.M. (2025). Managing adverse effects of incretin-based medications for obesity. JAMA.

[77] Anyiam O., Ardavani A., Rashid R.S.A., Panesar A., Idris I. (2025). How do glucagon-like peptide-1 receptor agonists affect measures of muscle mass in individuals with, and without, type 2 diabetes: a systematic review and meta-analysis. Obes Rev Off J Int Assoc Study Obes.

[78] Chen W., Qin H., Zhou Z. (2025). Glucagon-like peptide-1 receptor agonists and sarcopenia-related markers in diabetes: a systematic review and meta-analysis. Clin Nutr.

[79] Moscucci F., Baratta F., Pastori D. (2026). A narrative review on GLP-1 receptor agonists for obesity in older women: maximizing weight loss while preserving lean mass. Nutrients.

[80] Yang Y., He L., Han S. (2025). Sex differences in the efficacy of glucagon-like peptide-1 receptor agonists for weight reduction: a systematic review and meta-analysis. J Diabetes.

[81] Kim S.W., Kim R. (2020). The association between hormone therapy and sarcopenia in postmenopausal women: the Korea National Health and Nutrition Examination Survey, 2008-2011. Menopause.

[82] Österdahl M.F., Ni Lochlainn M., Welch C. (2025). Systematic review on the relationship between menopausal hormone replacement therapy, sarcopenia, and sarcopenia-related parameters. Maturitas.

[83] Javed A.A., Mayhew A.J., Shea A.K., Raina P. (2019). Association between hormone therapy and muscle mass in postmenopausal women: a systematic review and meta-analysis. JAMA Netw Open.

[84] Cortes T.M., Chae K., Foy C.M., Houston D.K., Beavers K.M. (2025). The impact of lifestyle-based weight loss in older adults with obesity on muscle and bone health: a balancing act. Obesity.

[85] American Diabetes Association Professional Practice Committee for Diabetes*. 13 (2026). Older adults: standards of care in diabetes—2026. Diabetes Care.

[86] Bohannon R.W. (2019). Grip strength: an indispensable biomarker for older adults. Clin Interv Aging.

[87] Mehmet H., Robinson S.R., Yang AWH. (2020). Assessment of gait speed in older adults. J Geriatr Phys Ther.

[88] Otadi K., Malmir K. (2026). Normative reference values for the six-minute walk test in older adults: a systematic review and meta-analysis. Arch Gerontol Geriatr.

[89] Pantazopoulos D., Gouveri E., Papazoglou D., Papanas N. (2025). GLP-1 receptor agonists and sarcopenia: weight loss at a cost? A brief narrative review. Diabetes Res Clin Pract.

[90] González-Luis A., Llinares-Arvelo V., CE Martínez-Alberto (2025). Glucagon-like peptide-1 receptor agonists and muscle health: potential role in sarcopenia prevention and treatment. Eur J Endocrinol.

[91] Bauer J., Biolo G., Cederholm T. (2013). Evidence-based recommendations for optimal dietary protein intake in older people: a position paper from the PROT-AGE Study Group. J Am Med Dir Assoc.

[92] Deutz N.E.P., Bauer J.M., Barazzoni R. (2014). Protein intake and exercise for optimal muscle function with aging: recommendations from the ESPEN Expert Group. Clin Nutr.

[93] Szwiega S., Pencharz P.B., Rafii M. (2021). Dietary leucine requirement of older men and women is higher than current recommendations. Am J Clin Nutr.

[94] Remelli F., Vitali A., Zurlo A., Volpato S. (2019). Vitamin D deficiency and sarcopenia in older persons. Nutrients.

[95] Giustina A., Bouillon R., Dawson-Hughes B. (2023). Vitamin D in the older population: a consensus statement. Endocrine.

[96] Jones M.D., Clifford B.K., Stamatakis E., Gibbs M.T. (2024). Exercise snacks and other forms of intermittent physical activity for improving health in adults and older adults: a scoping review of epidemiological, experimental and qualitative studies. Sports Med.

[97] Rolland Y., Dray C., Vellas B., Barreto PDS. (2023). Current and investigational medications for the treatment of sarcopenia. Metabolism.

[98] Cho M.R., Lee S., Song S.K. (2022). A review of sarcopenia pathophysiology, diagnosis, treatment and future direction. J Korean Med Sci.

[99] Nicholson K., Liu W., Fitzpatrick D. (2024). Prevalence of multimorbidity and polypharmacy among adults and older adults: a systematic review. Lancet Healthy Longev.

[100] Marassi M., Fadini G.P. (2026). GLP-1 receptor agonists in older people with type 2 diabetes: safety evidence from the real world. Expert Opin Drug Saf.

[101] Artaud F., Singh-Manoux A., Dugravot A., Tzourio C., Elbaz A. (2015). Decline in fast gait speed as a predictor of disability in older adults. J Am Geriatr Soc.

[102] Adam C.E., Fitzpatrick A.L., Leary C.S. (2023). Change in gait speed and fall risk among community-dwelling older adults with and without mild cognitive impairment: a retrospective cohort analysis. BMC Geriatr.

[103] Westbury L.D., Beaudart C., Bruyère O. (2023). Recent sarcopenia definitions—Prevalence, agreement and mortality associations among men: findings from population-based cohorts. J Cachexia Sarcopenia Muscle.

